# A Comparison of Serum and Plasma Blood Collection Tubes for the Integration of Epidemiological and Metabolomics Data

**DOI:** 10.3389/fmolb.2021.682134

**Published:** 2021-07-08

**Authors:** Jennie Sotelo-Orozco, Shin-Yu Chen, Irva Hertz-Picciotto, Carolyn M. Slupsky

**Affiliations:** ^1^ Department of Public Health Sciences, University of California Davis, Davis, CA, United States; ^2^ Department of Food Science and Technology, University of California Davis, Davis, CA, United States; ^3^ Department of Nutrition, University of California Davis, Davis, CA, United States

**Keywords:** metabolomics, metabolic profile, NMR spectroscopy, plasma, serum, anticoagulants, epidemiological studies

## Abstract

Blood is a rich biological sample routinely collected in clinical and epidemiological studies. With advancements in high throughput -omics technology, such as metabolomics, epidemiology can now delve more deeply and comprehensively into biological mechanisms involved in the etiology of diseases. However, the impact of the blood collection tube matrix of samples collected needs to be carefully considered to obtain meaningful biological interpretations and understand how the metabolite signatures are affected by different tube types. In the present study, we investigated whether the metabolic profile of blood collected as serum differed from samples collected as ACD plasma, citrate plasma, EDTA plasma, fluoride plasma, or heparin plasma. We identified and quantified 50 metabolites present in all samples utilizing nuclear magnetic resonance (NMR) spectroscopy. The heparin plasma tubes performed the closest to serum, with only three metabolites showing significant differences, followed by EDTA which significantly differed for five metabolites, and fluoride tubes which differed in eleven of the fifty metabolites. Most of these metabolite differences were due to higher levels of amino acids in serum compared to heparin plasma, EDTA plasma, and fluoride plasma. In contrast, metabolite measurements from ACD and citrate plasma differed significantly for approximately half of the metabolites assessed. These metabolite differences in ACD and citrate plasma were largely due to significant interfering peaks from the anticoagulants themselves. Blood is one of the most banked samples and thus mining and comparing samples between studies requires understanding how the metabolite signature is affected by the different media and different tube types.

## Introduction

Classical epidemiological studies seek to identify risk factors to determine the presence or absence of disease and health in a population. Given the technological advancements in high-throughput -omics, epidemiology is now in a more powerful position to be able to uncover biological mechanisms involved in the etiology of different diseases. While research in past decades has identified candidate metabolites contributing to diseases, metabolomics, in particular, offers an unprecedented opportunity to enhance an epidemiologist’s traditional toolbox as metabolites are the final products of the genomics, transcriptomics, and proteomics cascade and provide a chemical “snapshot” of an organism’s entire metabolic state at any given time.

A wide variety of biological specimens (urine, blood, cerebrospinal fluid, saliva, etc.) may be utilized for metabolomics analysis. Blood is a rich biological sample that is sensitive to the effects of health or disease, genetic variation, environment, nutrition, or the impact of toxicants and is easily obtained and commonly biobanked as serum and plasma in large repositories that collect, process, store, and distribute samples for future scientific investigations. Biobanks are important resources for studies of the connection between genes and diseases, response to drugs and treatments, and other outcomes related to understanding diseases. Several countries have established national and international biobanks as repositories for biological samples, including blood samples. Established in 2006, the United Kingdom (UK) Biobank (Elliott et al., 2008), for example, has collected EDTA plasma, acid citrate dextrose (ACD) plasma, and urine, among other biosamples for future use. In 2015, the National Institute of Health (NIH) initiated the Precision Medicine Initiative (PMI) All of Us Research Program (Sankar and Parker, 2017), which will be the largest longitudinal study in the United States with a cohort of one million volunteers. PMI All of Us aims to understand how a person’s genetics, environment, and lifestyle can help to determine the best approach to prevent or treat disease by collecting genetic data, health data, and biological samples (including serum, EDTA plasma, citrate plasma, heparin plasma). In 2020, NIH announced blood samples collected from PMI All of Us participants will be tested for severe acute respiratory syndrome coronavirus 2 (SARS-CoV-2) antibodies to track prior infections within the US population to address the unprecedented coronavirus disease 2019 (Covid-19) global pandemic—exemplifying the importance of biorepository samples. Metabolomics analysis could be used in a similar endeavor to understand how biomarkers are associated with disease progression, or how metabolites are related to molecular changes in treatment therapies, for instance (Shen et al., 2020). However, understanding whether and/or how the blood collection tube matrices (anticoagulants) affect measurement of the metabolome is critical to successfully merge epidemiology and metabolomics.

Serum is often considered the gold standard as it is obtained from blood that has been coagulated and requires no additives, whereas plasma is obtained by mixing blood with an anticoagulant to inhibit the blood from clotting, followed by collecting the plasma supernatant. While the choice of serum or plasma may depend on the specific research purpose, it may also depend on sample availability such as in clinical and epidemiological studies which routinely biobank biological samples for future analysis. Several previous studies have investigated differences in the metabolic profile based on blood collection tubes (Supplementary Table S1). However, most of the extant literature is limited to investigating EDTA and Heparin plasma (Teahan et al., 2006; Pereira et al., 2010; Bernini et al., 2011; Denery et al., 2011; Wedge et al., 2011; Yu et al., 2011; López-Bascón et al., 2016; Suarez-Diez et al., 2017; Cruickshank-Quinn et al., 2018; Liu et al., 2018; Nishiumi et al., 2018; Paglia et al., 2018), while a few studies have also investigated other plasma tubes [such as citrate plasma (Bernini et al., 2011), potassium oxalate (Yin et al., 2013)] or have done comparisons using animal models (Zhou et al., 2017). Given the assortment of other plasma tube types available and the range of potential blood samples available in biobanks, here we sought to compare how the NMR-based metabolic profiles of serum compared to those profiles from plasma collected in ACD tubes, citrate plasma tubes, EDTA plasma tubes, fluoride plasma tubes, and heparin plasma tubes under the same collection conditions. We aimed to clarify the compositional differences between serum and plasma across the various collection tubes.

## Materials and Methods

### Blood Samples

Blood samples were collected from eight healthy volunteers (22–40 years old, with a BMI between 20–25, all female) after an overnight fast. For each subject, serum and plasma samples were collected into six different tubes that included plastic tubes with no additives (for serum), and acid citrate dextrose plasma (ACD), sodium citrate (Citrate), ethylenediaminetetraacetic acid (EDTA), sodium fluoride (Fluoride), and sodium heparin (Heparin) (for plasma) (BD Biosciences, San Jose CA). Each plasma and serum sample was processed as designated by the manufacturer’s specification. Briefly, for plasma, collection tubes were inverted eight times followed by centrifugation at ≤1,300 RCF for 10 min at 20°C. Serum tubes were gently inverted five times followed by a 45–60 min resting period at room temperature to obtain complete coagulation before performing the same centrifugation process as for plasma. Each serum/plasma sample was aliquoted into three 1.0 ml fractions and stored at -80°C until metabolomics analysis. All volunteers gave their written informed consent prior to participation in the study. The study was approved by the institutional review board at the University of California, Davis.

### Nuclear Magnetic Resonance Experiment

Three analyses were performed (three independent sample preparations on separate days) on each sample as follows. All samples were prepared in the same laboratory, using the same standard protocols. Data were collected on the same instrument operating with the same settings, but on different days. Plasma and serum samples were thawed, then filtered through an Amicon 3,000 MW cut-off Centrifugal Device to remove lipids and proteins. A 207 µL aliquot of the water-soluble filtrate was collected and combined with 23 µL of an internal standard (ISTD) consisting of 4.47 mM DSS-D6 ([3-(trimethylsilyl)-1-propanesulfonic acid-d6], 0.2% NaN3, in 99.8% D2O). The pH of each sample was adjusted to 6.8+/- 0.1 by adding small amounts of NaOH or HCl. The volumes of added HCl and NaOH were recorded. A 180 µL aliquot of the mixture was transferred to a labeled 3 mm Bruker NMR tube and stored at 4°C until NMR acquisition (within 24 h of sample preparation). Samples were run on a 600 MHz Bruker AVANCE III NMR spectrometer equipped with a TCI cryoprobe and SampleJet autosampler using the NOESY-presaturation pulse sequence (noesypr). NMR spectra were acquired at 25°C, with water saturation of 2.5 s during the prescan delay, a mixing time of 100 ms, 12 ppm sweep width, an acquisition time of 2.5 s, eight dummy scans, and 32 transients. All spectra were zero-filled to 128K data points and Fourier transformed with a 0.5-Hz line broadening applied. Spectra were manually phased and baseline-corrected and metabolites were identified and quantified using NMR Suite v8.1 (Chenomx Inc., Edmonton, Canada) (Weljie et al., 2006). After analysis, a list of compounds together with their respective concentrations, based on the concentration of the added internal standard (DSS-d6), was generated. All compounds in the database have been verified against known concentrations of reference NMR spectra of the pure compounds and have been shown to be reproducible and accurate (Slupsky et al., 2007; Smilowitz et al., 2013). The metabolite concentrations (µM) of the three NMR experiments (technical replicates) were averaged for each sample to determine the final metabolite concentrations used in all analyses. Excellent reproducibility was shown for the three replicates (Table 1, Supplementary Figure S1).

**TABLE 1:** Blood metabolites measured using ^1^H NMR spectroscopy. Metabolites are listed in alphabetical order.

	ACD	Citrate	EDTA	Fluoride	Heparin	Serum	HMDB reference values^b^
	*(N = 8)*	*(N = 8)*	*(N = 8)*	*(N = 8)*	*(N = 8)*	*(N = 8)*	*(uM)*
2-Aminobutyrate							
Mean (SD)	22.4 (6.4)	22.7 (6.9)	24.0 (7.9)	24.8 (8.0)	24.3 (8.8)	24.4 (8.1)	23.0 (15.0–31.0)
							Cynober (2002)
Median (range)	23.7 (13.3–30.7)	23.4 (13.4–31.1)	25.7 (13.0–34.6)	25.5 (12.5–34.8)	25.2 (11.7–34.9)	25.3 (13.0–35.3)	
%CV (SD)^a^	4.3 (2.9)	5.4 (3.9)	7.4 (4.4)	5.4 (3.3)	7.2 (5.7)	6.8 (4.45)	
2-Hydroxybutyrate							
Mean (SD)	20.8 (6.8)	25.5 (8.8)	29.2 (10.4)	31.4 (10.3)	30.1 (8.7)	31.8 (10.1)	31.3 (7.8)
							Zordoky et al. (2015)
Median (range)	18.6 (15.6–35.9)	21.8 (18.3–45.3)	25.2 (21.5–52.7)	28.3 (22.0–55.0)	28.3 (21.5–48.5)	28.7 (23.6–54.8)	
%CV (SD)^a^	3.3 (2.5)	3.2 (1.7)	5.9 (3.3)	3.2 (0.8)	7.2 (6.5)	3.5 (3.0)	
2-Hydroxyisovalerate							
Mean (SD)	4.4 (1.7)	5.5 (2.1)	6.1 (2.7)	6.2 (2.9)	6.1 (2.9)	6.5 (3.1)	7.7 (0.0–19.0)
							Lentner (1981-1992)
Median (range)	3.7 (3.1–8.2)	4.8 (3.2–9.8)	4.9 (3.4–11.6)	5.2 (3.2–12.1)	5.0 (3.4–12.1)	5.5 (3.2–12.6)	
%CV (SD)^a^	6.9 (6.4)	10.8 (5.5)	7.3 (3.4)	7.4 (3.8)	8.4 (4.1)	4.9 (3.3)	
2-Oxoglutarate							
Mean (SD)	6.7 (1.5)	7.89 (1.9)	9.8 (2.3)	6.4 (2.7)	8.4 (2.1)	9.1 (2.4)	8.9 (2.7)
							Lentner (1981-1992)
Median (range)	6.7 (4.9–8.7)	7.3 (5.6–11.4)	9.2 (7.6–13.8)	5.9 (2.6–11.2)	7.8 (6.4–12.6)	9.1 (6.2–13.2)	
%CV (SD)^a^	7.1 (6.1)	9.9 (5.8)	7.0 (4.3)	10.5 (5.9)	7.6 (2.5)	9.2 (5.3)	
2-Oxoisocaproate							
Mean (SD)	3.7 (0.8)	4.6 (1.2)	4.8 (1.3)	5.2 (1.5)	5.2 (1.5)	5.4 (1.5)	28.0 (0.0–58.0)
							Hoffmann et al. (1993)
Median (range)	3.6 (2.7–5.3)	4.5 (3.2–6.9)	4.5 (3.7–7.6)	4.8 (3.7–8.3)	4.9 (3.7–8.2)	5.1 (3.8–8.2)	
%CV (SD)^a^	9.7 (5.6)	4.7 (1.5)	9.2 (6.5)	7.1 (4.8)	8.3 (3.8)	6.7 (4.6)	
3-Hydroxybutyrate							
Mean (SD)	58.1 (71.1)	60.0 (69.9)	83.1 (108.1)	83.1 (108.1)	71.7 (74.9)	74.1 (84.4)	76.9 (66.3)
							Psychogios et al. (2011)
Median (range)	31.1 (20.4–230.7)	30.8 (22.3–228.3)	37.8 (30.9–345.4)	39.6 (30.9–346.2)	38.5 (30.4–247.7)	38.1 (31.4–277.2)	
%CV (SD)^a^	4.7 (3.8)	2.9 (1.8)	5.4 (3.5)	2.9 (0.9)	7.9 (7.7)	4.4 (2.5)	
3-Hydroxyisobutyrate							
Mean (SD)	12.6 (2.1)	14.1 (2.2)	15.9 (2.3)	15.0 (2.0)	15.9 (2.4)	16.4 (2.3)	21.0 (2.0)
							Avogaro and Bier (1989)
Median (range)	11.8 (10.8–16.2)	13.3 (11.9–18.8)	15.5 (13.0–19.8)	15.2 (11.2–18.0)	15.5 (12.1–20.3)	16.0 (13.6–21.2)	
%CV (SD)^a^	5.3 (3.4)	3.0 (3.1)	5.4 (2.8)	4.6 (1.4)	7.1 (5.3)	3.9 (2.6)	
3-Methyl-2-oxobutanoate							
Mean (SD)	10.9 (2.1)	13.5 (3.1)	14.7 (3.5)	15.3 (4.0)	15.5 (4.3)	15.9 (3.7)	11.0 (1.7)
							Lentner (1981-1992)
Median (range)	10.7 (7.8–15.7)	13.6 (9.3–20.0)	14.4 (10.2–22.6)	15.0 (10.7–24.1)	14.5 (11.2–25.5)	15.3 (11.0–23.9)	
%CV (SD)^a^	4.2 (3.5)	2.4 (1.8)	5.0 (3.3)	4.4 (1.7)	6.2 (7.2)	4.0 (3.0)	
Acetate							
Mean (SD)	39.1 (8.7)	56.9 (12.6)	60.6 (14.6)	51.7 (14.3)	43.2 (13.1)	38.1 (9.1)	41.9 (15.1)
							Psychogios et al. (2011)
Median (range)	40.1 (25.3–51.0)	59.5 (38.6–74.6)	63.1 (41.5–81.6)	50.3 (36.7–78.2)	43.3 (27.5–62.7)	40.4 (25.5–50.3)	
%CV (SD)^a^	4.6 (3.7)	4.5 (3.2)	6.1 (4.3)	2.8 (1.4)	9.2 (6.9)	6.4 (1.9)	
Acetoacetate							
Mean (SD)	26.9 (23.9)	57.3 (20.4)	37.4 (37.1)	37.9 (38.1)	31.5 (20.9)	29.2 (21.0)	40.6 (36.5)
							Psychogios et al. (2011)
Median (range)	18.6 (9.3–84.4)	51.2 (41.3–105.3)	25.8 (14.0–127.1)	24.9 (15.0–130.3)	25.0 (13.5–78.3)	22.6 (13.2–78.3)	
%CV (SD)^a^	4.4 (4.7)	2.4 (2.1)	4.8 (2.8)	3.5 (2.6)	8.1 (7.0)	4.9 (2.5)	
Acetone							
Mean (SD)	33.8 (12.1)	115.0 (20.0)	20.6 (16.6)	18.6 (15.4)	19.2 (15.0)	20.49 (15.9)	54.4 (29.6)
							Psychogios et al. (2011)
Median (range)	29.7 (24.4–59.7)	121.7 (83.6–137.0)	14.0 (9.1–57.4)	13.7 (7.4–52.8)	15.0 (7.6–51.7)	15.0 (8.0–54.1)	
%CV (SD)^a^	7.6 (4.0)	4.9 (3.3)	8.8 (4.4)	8.8 (5.3)	9.7 (6.6)	8.2 (3.7)	
Alanine							
Mean (SD)	274.7 (44.7)	287.1 (51.7)	331.1 (63.3)	291.3 (52.6)	331.1 (65.8)	370.9 (60.5)	427.2 (84.4)
							Psychogios et al. (2011)
Median (range)	271.5 (195.0–339.6)	278.5 (207.5–356.4)	324.7 (232.6–431.1)	284.5 (204.7–372.1)	327.6 (218.0–421.5)	369.4 (272.1–460.4)	
%CV (SD)^a^	3.1 (3.5)	2.1 (0.8)	4.9 (2.8)	2.5 (1.5)	6.1 (5.7)	3.4 (2.0)	
Arginine							
Mean (SD)	59.9 (8.6)	60.4 (9.6)	64.2 (7.2)	64.3 (11.0)	64.9 (12.3)	78.1 (9.2)	113.6 (14.6)
							Psychogios et al. (2011)
Median (range)	61.7 (44.4–73.8)	62.4 (41.6–75.0)	61.1 (54.1–72.7)	62.8 (47.8–78.0)	67.1 (39.7–78.1)	80.4 (61.9–89.9)	
%CV (SD)^a^	8.5 (6.7)	10.7 (5.9)	12.1 (5.1)	12.7 (8.3)	11.2 (4.3)	6.5 (3.3)	
Asparagine							
Mean (SD)	38.7 (6.2)	42.1 (6.2)	48.9 (7.5)	42.3 (7.6)	48.7 (7.8)	52.8 (8.7)	41.0 (10.0)
							Psychogios et al. (2011)
Median (range)	41.9 (28.3–45.0)	40.2 (35.2–50.6)	49.5 (40.0–58.9)	45.0 (29.8–52.6)	50.5 (34.3–57.8)	54.9 (40.6–63.6)	
%CV (SD)^a^	7.3 (3.2)	8.7 (3.6)	11.4 (3.5)	7.6 (4.6)	6.2 (5.7)	5.5 (2.8)	
Betaine							
Mean (SD)	35.4 (11.3)	33.0 (11.5)	40.3 (14.6)	33.3 (12.1)	40.1 (14.9)	41.9 (14.8)	72.0 (22.4)
							Psychogios et al. (2011)
Median (range)	35.9 (13.2–51.6)	33.5 (11.2–50.4)	40.9 (15.4–65.1)	33.2 (12.1–53.9)	38.5 (15.4–66.6)	42.1 (15.2–67.1)	
%CV (SD)^a^	5.8 (3.4)	4.5 (3.6)	10.4 (5.3)	4.7 (1.8)	6.3 (5.6)	3.6 (3.2)	
Butyrate							
Mean (SD)	3.1 (0.6)	3.6 (0.7)	4.1 (0.7)	5.0 (1.2)	4.2 (1.1)	4.5 (0.9)	1.0 (0.3–1.5)
							Lentner (1981-1992)
Median (range)	2.9 (2.5–4.0)	3.7 (2.6–4.7)	4.0 (3.2–5.4)	4.8 (3.4–6.8)	4.2 (3.1–6.7)	4.4 (3.5–5.9)	
%CV (SD)^a^	12.3 (4.8)	6.7 (2.8)	10.1 (3.7)	8.7 (3.1)	11.2 (4.4)	8.3 (5.0)	
Carnitine							
Mean (SD)	33.6 (3.6)	28.1 (3.2)	34.6 (5.5)	29.6 (3.9)	33.4 (5.7)	34.3 (5.1)	45.7 (11.6)
							Psychogios et al. (2011)
Median (range)	32.7 (30.4–41.6)	26.2 (25.4–33.6)	35.9 (27.8–41.9)	29.1 (24.0–37.2)	31.6 (26.8–45.4)	32.6 (29.7–45.7)	
%CV (SD)^a^	4.1 (4.0)	3.2 (2.6)	11.1 (6.7)	3.6 (1.9)	7.8 (6.6)	5.7 (1.7)	
Choline							
Mean (SD)	6.6 (1.3)	6.2 (1.1)	9.6 (5.5)	7.6 (1.3)	7.3 (1.5)	8.1 (1.4)	14.5 (5.3)
							Psychogios et al. (2011)
Median (range)	6.3 (5.2–9.3)	6.1 (4.8–8.6)	7.6 (6.0–23.0)	7.2 (5.9–10.3)	7.1 (5.6–10.3)	7.8 (6.7–11.4)	
%CV (SD)^a^	6.0 (2.0)	7.9 (6.0)	15.3 (18.7)	4.0 (1.7)	9.5 (6.5)	4.1 (2.4)	
Citrate							
Mean (SD)	32,811.3 (2,920.0)	21,014.6 (1,592.1)	117.6 (30.2)	124.7 (34.9)	90.4 (27.2)	109.9 (32.4)	114.2 (27.0)
							Psychogios et al. (2011)
Median (range)	32,460.3 (28,837.0–37,319.6)	20,813.0 (18,344.4–22,967.5)	111.1 (66.7–162.7)	118.1 (69.7–184.5)	83.8 (49.2–129.8)	102.9 (60.0–167.1)	
%CV (SD)^a^	3.1 (3.3)	1.9 (1.8)	11.4 (4.6)	4.4 (1.9)	10.0 (6.3)	5.6 (4.0)	
Creatine							
Mean (SD)	27.7 (14.8)	27.1 (14.4)	34.5 (19.6)	30.3 (16.8)	33.1 (17.2)	34.9 (18.1)	36.7 (28.3)
							Psychogios et al. (2011)
Median (range)	24.0 (11.6–52.7)	24.8 (11.4–51.5)	29.2 (13.7–69.1)	26.5 (12.1–59.7)	30.1 (12.5–64.1)	31.3 (14.4–65.1)	
%CV (SD)^a^	5.1 (3.3)	3.6 (2.3)	6.1 (2.9)	4.8 (1.7)	7.0 (6.0)	4.3 (2.3)	
Creatinine							
Mean (SD)	51.9 (9.8)	55.1 (11.1)	63.1 (13.5)	58.8 (13.9)	63.3 (13.0)	65.8 (13.2)	86.6 (18.8)
							Psychogios et al. (2011)
Median (range)	56.6 (36.3–64.6)	61.0 (41.0–66.9)	67.4 (44.1–82.6)	65.1 (38.7–75.6)	66.5 (47.6–82.1)	71.7 (46.5–82.3)	
%CV (SD)^a^	3.2 (3.7)	2.5 (1.9)	5.9 (3.6)	3.6 (1.6)	7.4 (6.3)	3.5 (1.9)	
Dimethyl sulfone							
Mean (SD)	5.6 (1.6)	6.1 (1.9)	7.2 (1.6)	6.9 (1.9)	6.8 (1.8)	7.3 (2.2)	8.8 (7.3)
							Engelke et al. (2005)
Median (range)	5.2 (4.0–8.0)	5.5 (4.2–9.2)	6.6 (5.8–9.7)	6.6 (4.7–9.9)	6.4 (4.7–9.8)	6.8 (4.8–10.4)	
%CV (SD)^a^	4.7 (3.8)	4.2 (2.6)	7.1 (2.9)	5.8 (3.9)	8.1 (5.9)	6.6 (2.7)	
Formate							
Mean (SD)	135.3 (5.8)	36.9 (2.3)	183.6 (7.8)	13.5 (2.3)	13.4 (1.8)	12.2 (1.9)	32.8 (3.3)
							Psychogios et al. (2011)
Median (range)	134.5 (125.9–143.9)	37.0 (33.4–40.3)	181.0 (174.4–198.9)	13.3 (10.9–18.7)	12.9 (11.4–17.1)	12.6 (9.9–15.1)	
%CV (SD)^a^	3.3 (3.4)	4.6 (1.6)	4.8 (3.3)	10.4 (5.9)	7.9 (4.6)	6.9 (4.5)	
Glucose							
Mean (SD)	22,764.9 (1,021.3)	4,134.3 (323.0)	4,712.0 (484.5)	4,505.2 (415.1)	4,505.0 (326.2)	4,511.9 (319.6)	4,971.3 (327.8)
							Psychogios et al. (2011)
Median (range)	22,686.5 (21,345.6–24,020.9)	4,039.6 (3,856.2–4,872.4)	4,653.4 (4,059.2–5,556.5)	4,357.5 (4,022.1–5,292.6)	4,434.0 (4,063.4–5,114.2)	4,525.9 (4,087.4–5,090.4)	
%CV (SD)^a^	3.2 (3.7)	3.2 (1.8)	4.9 (3.0)	3.6 (2.0)	6.9 (6.7)	3.3 (3.2)	
Glutamate							
Mean (SD)	41.0 (11.7)	23.6 (6.8)	38.5 (14.7)	34.1 (13.1)	30.2 (9.5)	44.3 (7.7)	97.4 (13.2)
							Psychogios et al. (2011)
Median (range)	41.2 (23.9–57.5)	21.1 (16.7–34.4)	36.6 (25.4–70.3)	32.1 (21.8–63.4)	34.0 (16.9–40.6)	45.4 (33.5–54.7)	
%CV (SD)^a^	10.1 (5.6)	8.0 (2.6)	10.2 (5.6)	12.0 (10.2)	9.9 (8.9)	10.5 (5.1)	
Glutamine							
Mean (SD)	415.7 (53.6)	436.0 (50.4)	482.4 (54.4)	436.9 (53.6)	492.6 (69.9)	516.7 (63.7)	510.4 (118.2)
							Psychogios et al. (2011)
Median (range)	435.7 (330.8–474.7)	445.4 (354.8–506.9)	486.1 (390.1–571.4)	445.8 (336.5–502.5)	504.2 (354.7–570.8)	528.3 (408.5–598.2)	
%CV (SD)^a^	5.5 (3.1)	5.5 (2.6)	5.6 (3.8)	4.5 (2.9)	6.2 (5.2)	4.0 (2.4)	
Glycine							
Mean (SD)	184.7 (48.7)	193.1 (55.8)	243.3 (62.3)	201.4 (56.4)	225.3 (67.4)	251.7 (64.1)	325.4 (126.8)
							Psychogios et al. (2011)
Median (range)	176.0 (137.6–291.6)	180.7 (136.3–317.2)	225.8 (189.6–387.8)	185.3 (148.5–329.1)	206.6 (174.8–375.5)	237.1 (192.8–396.1)	
%CV (SD)^a^	3.4 (2.8)	2.2 (1.6)	4.6 (3.1)	2.2 (1.2)	6.2 (5.8)	3.3 (2.1)	
Histidine							
Mean (SD)	51.1 (5.8)	56.8 (5.5)	64.6 (8.8)	49.3 (9.2)	58.7 (9.5)	63.7 (9.6)	131.2 (37.2)
							Psychogios et al. (2011)
Median (range)	54.2 (43.6–56.5)	57.2 (50.0–66.0)	62.7 (55.4–79.4)	47.2 (39.6–64.2)	61.0 (42.8–73.9)	62.5 (50.4–76.9)	
%CV (SD)^a^	8.7 (3.6)	7.0 (4.5)	10.2 (3.2)	12.9 (8.5)	10.4 (5.4)	6.5 (3.6)	
Isoleucine							
Mean (SD)	51.1 (9.9)	54.8 (10.0)	60.5 (13.2)	60.4 (13.4)	60.1 (13.7)	63.7 (12.4)	60.7 (18.6)
							Psychogios et al. (2011)
Median (range)	48.4 (41.0–66.5)	53.5 (44.9–71.4)	55.7 (47.8–82.1)	58.5 (47.4–81.5)	58.5 (43.9–80.4)	60.5 (51.9–85.2)	
%CV (SD)^a^	4.4 (3.6)	2.3 (1.8)	5.1 (3.1)	3.0 (2.2)	6.7 (6.3)	3.7 (2.5)	
Lactate							
Mean (SD)	885.1 (322.7)	1,041.8 (419.5)	1,635.2 (583.1)	1,194.4 (549.3)	1,547.7 (610.3)	2,170.8 (520.0)	1,489.4 (371.2)
							Psychogios et al. (2011)
Median (range)	830.8 (401.5–1,399.4)	972.2 (623.3–1972.7)	1,338.4 (997.2–2,505.5)	997.9 (440.9–2092.1)	1,293.5 (1,042.6–2,734.9)	2038.7 (1,528.1–3,219.3)	
%CV (SD)^a^	4.4 (3.8)	5.0 (3.4)	5.9 (3.0)	3.1 (1.6)	7.5 (5.5)	3.1 (3.2)	
Leucine							
Mean (SD)	78.2 (15.6)	79.5 (14.8)	91.1 (20.4)	90.6 (22.1)	94.5 (22.4)	99.9 (20.3)	98.7 (11.5)
							Psychogios et al. (2011)
Median (range)	73.2 (64.4–106.7)	78.5 (62.5–105.7)	86.1 (73.4–124.7)	86.7 (68.6–127.6)	94.5 (69.4–134.3)	95.6 (80.3–132.8)	
%CV (SD)^a^	4.7 (3.9)	3.2 (1.6)	5.2 (3.3)	3.8 (2.7)	6.7 (6.0)	4.3 (2.0)	
Lysine							
Mean (SD)	107.9 (23.2)	113.1 (24.1)	132.1 (30.8)	115.4 (25.7)	127.5 (30.2)	137.2 (28.5)	178.6 (58.2)
							Psychogios et al. (2011)
Median (range)	108.8 (74.7–140.0)	109.2 (76.7–145.8)	133.1 (82.0–169.4)	115.1 (75.9–145.3)	127.2 (79.4–159.5)	137.9 (91.6–172.8)	
%CV (SD)^a^	4.8 (2.8)	4.9 (2.5)	6.4 (4.7)	4.3 (1.8)	5.6 (5.3)	4.7 (3.1)	
Mannose							
Mean (SD)	40.6 (6.2)	44.5 (7.9)	47.6 (6.0)	47.6 (8.9)	47.7 (7.1)	45.9 (7.0)	39.0 (7.0)
							Lentner (1981-1992)
Median (range)	39.8 (31.0–51.6)	47.9 (30.6–52.3)	48.6 (36.3–55.0)	49.7 (34.6–60.5)	49.6 (35.7–56.7)	47.6 (34.8–54.6)	
%CV (SD)^a^	8.5 (5.0)	10.0 (5.3)	7.6 (3.7)	6.9 (4.5)	8.5 (4.2)	7.9 (4.5)	
Methanol							
Mean (SD)	68.2 (15.0)	29.4 (15.8)	27.9 (17.8)	28.1 (16.8)	27.7 (15.1)	28.0 (18.5)	77.4 (16.3)
							Psychogios et al. (2011)
Median (range)	65.9 (52.3–99.6)	28.0 (12.1–60.0)	26.1 (11.2–65.0)	26.9 (11.0–59.7)	27.8 (11.3–55.3)	26.6 (8.4–63.9)	
%CV (SD)^a^	4.9 (4.9)	5.9 (5.1)	9.8 (4.8)	4.8 (1.8)	9.3 (5.9)	4.8 (3.7)	
Methionine							
Mean (SD)	18.0 (2.3)	20.7 (2.9)	24.0 (3.7)	23.9 (3.7)	24.3 (3.5)	25.6 (3.4)	29.8 (6.3)
							Psychogios et al. (2011)
Median (range)	17.6 (16.3–23.5)	19.6 (18.0–27.2)	23.8 (18.7–31.1)	22.5 (20.2–31.8)	24.1 (20.2–31.9)	24.7 (22.7–33.5)	
%CV (SD)^a^	6.1 (4.2)	9.8 (4.8)	9.2 (5.3)	5.0 (3.2)	6.5 (4.8)	7.3 (4.8)	
Myo inositol							
Mean (SD)	18.3 (2.1)	18.3 (1.8)	22.9 (2.1)	18.8 (2.6)	23.9 (2.0)	25.6 (3.0)	23.0 (8.0)
							Psychogios et al. (2011)
Median (range)	18.5 (15.2–21.3)	18.8 (15.6–20.8)	21.9 (20.8–26.0)	19.2 (15.0–22.9)	23.9 (20.6–26.3)	26.6 (21.0–29.0)	
%CV (SD)^a^	4.6 (4.8)	9.6 (4.9)	12.0 (2.8)	14.1 (9.6)	11.9 (4.9)	11.2 (5.2)	
N,N-Dimethylglycine							
Mean (SD)	1.8 (0.5)	1.9 (0.6)	2.2 (0.7)	2.1 (0.5)	2.2 (0.7)	2.3 (0.7)	2.6 (1.8–3.7)
							Mcgregor et al. (2001)
Median (range)	1.7 (1.3–2.8)	1.8 (1.2–2.8)	2.1 (1.3–3.5)	2.0 (1.4–3.1)	2.1 (1.6–3.6)	2.2 (1.6–3.6)	
%CV (SD)^a^	5.8 (4.4)	10.3 (4.4)	9.6 (7.3)	7.9 (4.8)	7.2 (3.8)	8.1 (4.4)	
O-Acetylcarnitine							
Mean (SD)	5.8 (1.3)	6.1 (1.5)	7.9 (2.1)	7.3 (1.3)	7.9 (1.3)	8.1 (1.5)	5.48 (2.15)
							Psychogios et al. (2011)
Median (range)	5.8 (4.2–8.1)	5.8 (4.3–8.2)	7.4 (6.0–11.3)	7.0 (5.8–9.5)	7.9 (5.3–9.6)	8.0 (6.4–10.3)	
%CV (SD)^a^	6.2 (3.6)	10.7 (6.1)	10.2 (5.6)	10.6 (5.6)	9.5 (4.8)	13.1 (5.9)	
Ornithine							
Mean (SD)	35.1 (8.2)	38.6 (9.2)	50.2 (12.5)	47.2 (11.4)	49.3 (14.3)	63.0 (15.3)	66.9 (15.3)
							Psychogios et al. (2011)
Median (range)	32.3 (22.5–48.3)	38.8 (23.9–52.6)	50.4 (30.5–71.9)	45.7 (30.9–64.5)	50.1 (27.0–73.1)	61.9 (39.1–93.9)	
%CV (SD)^a^	6.9 (3.7)	4.3 (2.6)	7.4 (3.6)	9.4 (5.9)	6.5 (5.5)	3.9 (2.4)	
Phenylalanine							
Mean (SD)	41.2 (6.1)	43.1 (5.7)	46.5 (7.1)	45.4 (7.7)	45.6 (7.7)	53.3 (6.8)	78.1 (20.5)
							Psychogios et al. (2011)
Median (range)	39.0 (36.6–54.4)	40.8 (38.4–54.8)	43.6 (41.2–61.9)	43.8 (37.7–61.4)	44.6 (37.4–63.5)	51.0 (48.1–68.6)	
%CV (SD)^a^	4.9 (3.1)	2.6 (1.2)	5.5 (3.1)	3.2 (2.2)	6.8 (7.3)	4.0 (1.3)	
Proline							
Mean (SD)	127.8 (32.9)	135.5 (30.1)	158.1 (42.6)	146.3 (40.4)	156.4 (42.6)	169.4 (44.4)	198.3 (64.8)
							Psychogios et al. (2011)
Median (range)	125.2 (75.4–175.3)	133.5 (81.4–174.9)	153.6 (93.1–231.4)	136.0 (85.7–213.8)	146.7 (88.6–221.7)	164.9 (97.4–235.4)	
%CV (SD)^a^	5.7 (3.3)	5.0 (4.3)	7.8 (3.7)	7.4 (4.4)	6.6 (5.5)	4.2 (2.4)	
Pyruvate							
Mean (SD)	89.6 (19.7)	80.7 (22.6)	110.4 (28.5)	6.0 (1.6)	76.8 (30.6)	72.2 (28.3)	64.0 (22–258)
							Psychogios et al. (2011)
Median (range)	93.2 (60.0–118.9)	75.0 (57.4–125.5)	109.5 (79.9–166.0)	5.4 (4.6–9.7)	73.2 (39.0–141.7)	69.0 (43.7–123.0)	
%CV (SD)^a^	3.9 (3.7)	4.7 (3.1)	5.5 (3.6)	5.9 (1.5)	7.0 (5.1)	3.1 (2.2)	
Serine							
Mean (SD)	96.9 (19.7)	95.8 (20.4)	113.5 (24.2)	101.0 (23.6)	112.6 (27.1)	133.1 (27.2)	159.8 (26.6)
							Psychogios et al. (2011)
Median (range)	95.8 (70.5–123.7)	90.6 (69.7–129.0)	105.8 (86.8–153.8)	94.7 (67.8–133.0)	101.2 (81.7–157.4)	126.0 (94.3–174.1)	
%CV (SD)^a^	5.2 (2.7)	5.4 (3.1)	6.7 (4.3)	5.4 (1.7)	5.8 (4.6)	3.3 (2.2)	
Succinate							
Mean (SD)	9.0 (1.4)	6.4 (2.5)	6.0 (2.1)	5.1 (1.9)	5.6 (2.2)	6.4 (2.7)	16.0 (0.0–32.0)
							Psychogios et al. (2011)
Median (range)	8.6 (7.5–12.2)	5.8 (3.8–10.8)	5.4 (3.7–10.0)	4.6 (3.3–9.3)	4.9 (3.3–10.0)	5.4 (4.0–11.5)	
%CV (SD)^a^	4.4 (4.2)	9.0 (5.5)	10.2 (5.0)	7.2 (6.4)	9.8 (5.4)	8.1 (4.4)	
Taurine							
Mean (SD)	133.3 (18.6)	55.4 (11.6)	59.0 (11.0)	65.9 (13.3)	67.2 (9.3)	146.5 (10.0)	141.0 (57.0)
							Lentner (1981-1992)
Median (range)	132.2 (108.2–170.5)	56.7 (37.9–71.8)	61.7 (40.7–70.0)	65.0 (43.6–83.2)	68.9 (51.5–77.2)	145.5 (134.6–167.5)	
%CV (SD)^a^	5.8 (2.6)	12.1 (7.5)	12.7 (4.9)	9.0 (7.5)	8.1 (6.0)	6.3 (4.6)	
Threonine							
Mean (SD)	93.6 (18.1)	97.9 (17.2)	114.2 (23.1)	104.9 (20.5)	111.4 (24.7)	118.3 (23.5)	127.7 (41.0)
							Psychogios et al. (2011)
Median (range)	100.1 (56.7–114.4)	96.4 (63.2–118.3)	115.6 (69.5–142.0)	107.9 (59.9–127.6)	116.5 (63.2–139.7)	123.7 (68.6–144.7)	
%CV (SD)^a^	8.2 (5.2)	6.6 (5.0)	7.6 (2.9)	5.8 (2.6)	7.0 (4.1)	5.6 (3.9)	
Tryptophan							
Mean (SD)	5.9 (1.2)	4.0 (1.3)	4.7 (0.8)	4.2 (1.1)	4.6 (1.0)	5.4 (1.0)	54.5 (9.7)
							Psychogios et al. (2011)
Median (range)	6.0 (4.6–7.7)	3.9 (1.9–6.2)	4.9 (3.0–5.7)	4.1 (2.8–5.5)	4.6 (2.9–6.2)	5.4 (3.9–7.3)	
%CV (SD)^a^	8.0 (5.1)	10.2 (9.1)	6.1 (4.0)	12.4 (17.1)	11.8 (5.3)	10.8 (5.2)	
Tyrosine							
Mean (SD)	51.8 (7.9)	54.1 (7.7)	60.1 (10.2)	58.3 (9.7)	59.2 (11.1)	63.5 (9.4)	54.5 (9.7)
							Psychogios et al. (2011)
Median (range)	50.8 (39.8–67.2)	54.1 (42.3–68.3)	58.4 (45.4–79.2)	57.1 (45.0–77.3)	58.6 (41.5–79.7)	61.4 (49.6–81.9)	
%CV (SD)^a^	4.1 (4.7)	2.2 (1.8)	6.1 (2.2)	3.6 (1.7)	5.6 (6.0)	2.9 (1.5)	
Urea							
Mean (SD)	1,663.5 (507.7)	1929.5 (466.1)	2,510.7 (688.1)	2,613.6 (788.1)	2,715.3 (790.9)	3,038.1 (722.4)	6,074.6 (2,154.2)
							Psychogios et al. (2011)
Median (range)	1,487.2 (1,097.2–2,572.7)	1715.9 (1,429.3–2,700.2)	2,225.3 (1809.4–3,458.5)	2,422.8 (1778.4–3,861.3)	2,525.5 (1904.4–3,731.2)	2,772.3 (2,317.5–3,971.9)	
%CV (SD)^a^	5.9 (3.0)	6.5 (4.4)	7.0 (4.0)	13.2 (6.8)	6.9 (3.3)	12.5 (13.1)	
Valine							
Mean (SD)	185.3 (27.6)	198.1 (30.1)	223.9 (39.0)	220.1 (39.3)	221.3 (42.6)	236.7 (36.6)	212.3 (61.3)
							Psychogios et al. (2011)
Median (range)	180.0 (146.7–231.4)	194.8 (157.7–249.4)	213.4 (171.2–290.4)	213.3 (170.8–283.5)	217.7 (158.5–285.0)	229.0 (190.1–299.2)	
%CV (SD)^a^	3.1 (3.5)	2.2 (1.8)	5.1 (3.0)	2.7 (2.2)	6.6 (6.8)	3.6 (2.1)	

a Percent coefficient of variation (%CV) was calculated as the mean (SD (%)) of the %CV for 48 samples (six tube types across eight subjects) that were individually prepared and analyzed in triplicate (three separate technical replicates) by ^1^H NMR (for a total of 144 samples).

b Human Metabolome Database (HMDB) reference values presented as mean (SD) where available or mean (range).

### Statistical Analysis

Metabolite concentrations were log10 transformed, and principal component analysis (PCA) was performed using the “prcomp” function where each variable was centered by subtracting to the variable means (center = True) but not scaled to the standard deviation (scale = FALSE) using the ggplot2 library in R. The mean difference and percent coefficient of variation (%CV defined as the standard deviation/mean × 100) of raw metabolite concentrations between serum (control) and each plasma tube type (ACD, Citrate, EDTA, Fluoride, or Heparin) were calculated to compare differences in specific metabolite concentrations between tubes. The Mann-Whitney U test was utilized to evaluate the significance of those differences, because not all metabolites followed a normal distribution. To account for multiple testing, we adjusted *p* values by controlling the false discovery rate (FDR) at 5% using the Benjamini-Hochberg procedure (p.adjust.method = “BH”) with *p*-values, 0.05 as statistically significant. Effect size between serum and each plasma tube was calculated using Cliff’s delta (δ) statistic (cliff.delta function from the effsize package). A |δ| < 0.33 corresponds to small, |δ| < 0.474 corresponds to medium, and |δ| > 0.475 corresponds to large effect size in metabolite concentration differences. The methodological precision for each metabolite was calculated as the mean ± SD (%) of the %CV for the 48 samples (six tube types tested across eight subjects) that were individually prepared and analyzed in triplicate by ^1^H NMR (for a total of 144 samples). Statistical computing and graphical generation were performed using the R programming environment. The identity of each sample was unblinded only after the analysis was completed. Literature derived reference values from the Human Metabolome Database (HMDB) are also presented for each metabolite to provide readers a better estimate of the potential concentration variations (Table 1).

## Results

A total of 52 metabolites were identified and quantified in our study. However, the metabolites cis-aconitate (which was only identified in ACD tubes) and ascorbate (which fell below the limit of detection for EDTA tubes) were excluded from any further analysis as they were not present in all tube types (Supplementary Figure S2). Therefore, a total of 50 metabolites were identified in all collection tubes and used in the analysis. These included amino acids and their metabolites (2-aminobutyrate, 2-hydroxybutyrate, 2-hydroxyisovalerate, 2-oxoisocaproate, 3-hydroxyisobutyrate, 3-methyl-2-oxobutanoate, alanine, arginine, asparagine, betaine, glutamate, glutamine, histidine, isoleucine, leucine, lysine, methionine, N,N-dimethylglycine, ornithine, phenylalanine, proline, serine, taurine, threonine, tryptophan, tyrosine, and valine), ketone bodies (3-hydroxybutyrate, acetoacetate, acetone), pyruvate metabolism (lactate and pyruvate), short-chain fatty acids (acetate and butyrate), sugars (glucose, mannose, and myo-inositol), tricarboxylic acid cycle metabolites (2-oxoglutarate, citrate, and succinate), creatine, creatinine, choline, dimethyl sulfone, methanol, and urea. The mean (SD) and median (interquartile range) of the metabolites, as well as the average %CV (SD) of the technical replicates for each tube type, are provided in Table 1. The average %CV ranged from 2 to 15% overall metabolites and tube types (average 7%), indicating a high degree of repeatability for sample preparation, data acquisition, and data analysis amongst all tube types.

Furthermore, we found that 31 out of 50 metabolite serum concentrations exhibited excellent agreement with concentrations reported in the literature (i.e., fell within one standard deviation of the literature value). An additional 11 metabolites fell within two standard deviations or reference range reported. However, not all our metabolite concentrations agree with literature-derived values. The greatest discrepancy between our serum measured values and the literature-derived values include: 3-hydroxyisobutyrate, 3-methyl-2-oxobutanoate, arginine, butyrate, formate, glutamate, methanol, and tryptophan. Although we have attempted to find reference values reported in the literature collected in a similar manner (i.e., NMR-derived serum values reported in healthy adult populations) some discrepancies between our values and those in the literature may be due to different analytical methods utilized (NMR versus mass-spectrometry), other anticoagulants utilized, differences in the study population, or possibly sample size effects. Nonetheless, 42 of the 50 metabolites (84%) exhibited good agreement with literature derived values.

**FIGURE 1:**
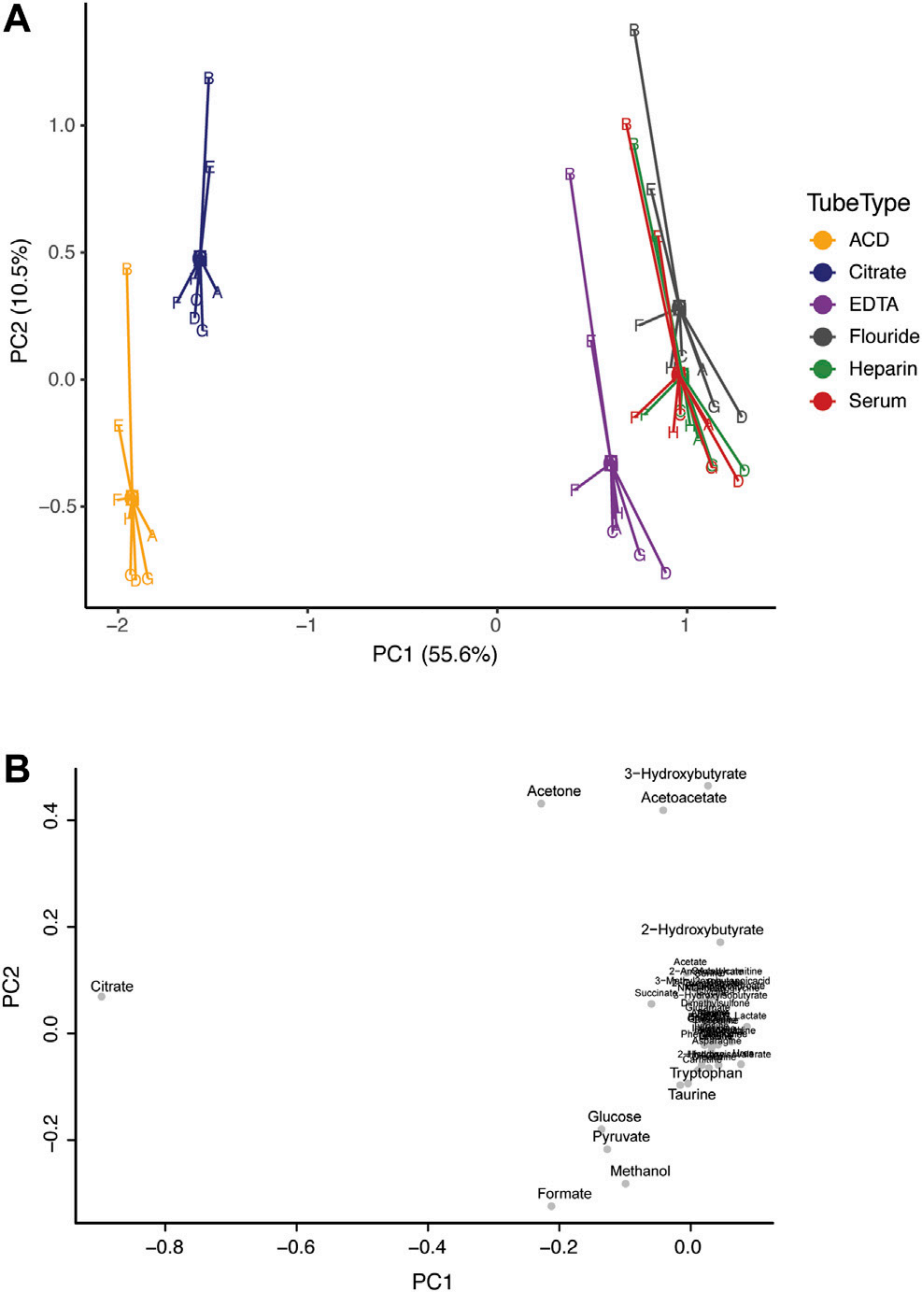
Comparison of the metabolic profile in serum and plasma samples from eight individuals. Subjects are represented by letters (A–H), and color indicates tube-type according to the legend **(A)** Principal component analysis (PCA) centroid plot of samples and **(B)** the corresponding loadings plot.

Principal component analysis (PCA) was applied to investigate inherent patterns in the metabolomic profiles (Figure 1). On the scores plot, each point represents a sample, with the same color representing the same tube type, and the same letter representing the same subject. The loadings plot indicates the contribution of the measured metabolites to the principal components. On the scores plot, principal component 1 (PC1) accounted for 55.6% of the variation and PC2 accounted for 10.5% of the variation. The corresponding loadings plot identified that citrate concentration greatly contributed to separation along PC1. Citrate is an additive in citrate tubes and in ACD plasma tubes (which also contain high levels of glucose). As such, the concentrations of citrate (in citrate plasma and ACD plasma), and glucose (in ACD plasma) do not reflect true biological concentrations (Supplementary Figure S3). However, there is a clear overlap in the metabolic profiles of all blood collection tube types, particularly for serum and heparin plasma. Additionally, differences between subjects were apparent along PC2, showing that subjects B and E tended to cluster toward the top of the plot regardless of tube type.

The heparin plasma tubes performed the closest to serum, with only three metabolites showing significant differences, followed by EDTA which significantly differed for five. Specifically, only 3 of 50 metabolites (6%), all of which were amino acids (arginine, glutamate, and taurine), were higher in serum compared to Heparin plasma. EDTA plasma differed in only 5 of 50 metabolites (10%) as compared to serum, which included higher levels of the amino acids arginine and taurine, and lower levels of pyruvate, acetate, and formate in serum compared to EDTA plasma. We also found that 11 of 50 metabolites (22%) were higher in serum compared to Fluoride plasma. These metabolites included amino acids (alanine, arginine, glutamate, glutamine, glycine, histidine, ornithine, taurine), as well as lactate, pyruvate, and myo-inositol. In contrast, metabolite measurements from plasma ACD tubes differed significantly for more than half of the 50 metabolites assessed. Specifically, ACD plasma varied in 29 of the 50 metabolites (58%) compared to serum. Interestingly, most of these metabolites had higher concentrations in serum compared to ACD plasma, which included amino acids and their metabolites, as well as o-acetylcarnitine, choline, urea, lactate, butyrate, and myo-inositol. As expected, glucose and citrate (both of which are additives in ACD plasma) were much lower in serum than in ACD tubes. Succinate, however, had a lower concentration in serum as compared to ACD plasma. Similar to ACD plasma, a high proportion (24 of 50 metabolites, 48%) were also significantly different in citrate plasma. Again, amino acids and their metabolites had increased levels in serum compared to citrate plasma, as well as carnitine, urea, lactate, glucose, and myo-inositol. In contrast, acetoacetate, acetone, acetate, and as expected, citrate concentrations were significantly lower in serum compared to citrate plasma tubes. The mean differences in the concentration of serum metabolites versus plasma samples are summarized in Table 2.

**TABLE 2:** Comparison of Serum Vs. Plasma. The table shows the mean difference, standard error of the mean (SEM), CV%, *p*-value, and effect size difference of metabolite concentrations (µM) in serum (reference) Vs. each respective plasma type.

Class	Metabolite	Serum vs. ACD					Serum vs. citrate					Serum vs. EDTA					Serum vs. fluoride					Serum vs. heparin				
		Mean diff	SEM	%CV	*p*-value^a^	Effect size ^b^	Mean diff	SEM	%CV	*p*-value^a^	Effect size ^b^	Mean diff	SEM	%CV	*p*-value^a^	Effect size ^b^	Mean diff	SEM	%CV	*p*-value^a^	Effect size ^b^	Mean diff	SEM	%CV	*p*-value^a^	Effect size ^b^
Amino Acids and their metabolites	*2-Aminobutyrate*	2.1	0.6	87.1	0.624	0.22	1.7	0.6	100.7	0.687	0.18	0.5	0.4	241.3	0.935	0.06	−0.3	3.9	−3,356.8	0.959	−0.04	0.2	0.6	980.6	0.935	0.06
	*2-Hydroxybutyrate*	11.0	1.3	32.7	0.027	1.52	6.4	0.6	27.9	0.126	0.80	2.6	0.3	36.8	0.781	0.33	0.4	2.2	1,527.1	0.959	0.05	1.8	0.8	121.0	0.858	0.20
	*2-Hydroxyisovalerate*	2.1	0.5	66.1	0.167	0.91	1.0	0.3	93.7	0.332	0.36	0.5	0.2	90.1	0.781	0.17	0.3	1.6	1,460.7	0.632	0.11	0.5	0.1	86.5	0.858	0.16
	*2-Oxoisocaproate*	1.7	0.3	45.2	0.031	1.51	0.8	0.2	50.4	0.373	0.63	0.5	0.1	50.2	0.831	0.39	0.2	0.8	1,255.0	0.784	0.14	0.2	0.1	136.1	0.887	0.14
	*3-Hydroxyisobutyrate*	3.8	0.4	26.7	0.027	1.82	2.4	0.2	22.1	0.086	1.09	0.5	0.3	178.9	0.879	0.22	1.4	0.7	129.2	0.513	0.66	0.5	0.2	106.9	0.858	0.24
	*3-Methyl-2-oxobutanoate*	5.1	0.6	33.6	0.019	1.83	2.4	0.3	35.7	0.186	0.74	1.2	0.3	65.3	0.781	0.35	0.6	2.1	953.6	0.887	0.19	0.4	0.4	251.0	0.858	0.13
	*Alanine*	96.2	6.8	19.9	0.023	1.78	83.9	4.6	15.5	0.039	1.47	39.8	4.3	30.9	0.696	0.65	79.6	29.2	103.7	0.141	1.38	39.8	4.6	32.8	0.858	0.63
	*Arginine*	18.2	2.6	39.6	0.018	1.96	17.7	2.2	34.6	0.021	1.75	13.9	3.8	77.6	0.148	1.65	13.8	6.3	129.7	0.141	1.33	13.2	3.5	75.7	0.341	1.12
	*Asparagine*	14.1	1.3	27.0	0.018	1.81	10.7	1.8	47.0	0.047	1.39	3.9	0.9	65.9	0.781	0.46	10.5	3.6	95.8	0.191	1.23	4.1	1.4	96.8	0.858	0.47
	*Betaine*	6.4	1.5	64.3	0.370	0.37	8.9	1.3	41.6	0.332	0.55	1.6	0.6	117.2	0.879	0.09	8.6	6.6	219.2	0.434	0.53	1.9	0.9	134.7	0.887	0.11
	*Glutamate*	3.3	2.6	219.7	0.562	0.41	20.7	2.3	31.9	0.005	2.83	5.8	3.2	156.3	0.781	0.67	10.2	4.6	127.0	0.141	1.13	14.1	1.5	30.8	0.174	1.49
	*Glutamine*	101.1	9.9	27.8	0.027	1.66	80.7	5.9	20.7	0.047	1.37	34.4	9.3	76.8	0.669	0.55	79.9	31.7	112.1	0.141	1.30	24.1	9.6	112.0	0.858	0.36
	*Glycine*	66.9	5.7	23.9	0.031	1.35	58.6	3.4	16.6	0.047	1.13	8.4	1.9	63.3	0.869	0.15	50.3	34.2	192.1	0.141	0.97	26.4	3.4	36.8	0.812	0.49
	*Histidine*	12.6	2.2	49.1	0.031	1.59	6.9	2.1	85.7	0.263	0.86	−0.9	0.9	-284.3	0.879	−0.12	14.4	5.8	112.8	0.141	1.55	5.0	1.9	108.3	0.858	0.52
	*Isoleucine*	12.6	1.1	25.1	0.096	1.18	8.9	1.1	34.6	0.223	0.82	3.1	0.6	55.3	0.869	0.28	3.3	7.5	652.2	0.631	0.29	3.6	0.9	73.0	0.858	0.31
	*Leucine*	21.7	1.9	24.4	0.050	1.28	20.4	2.3	31.2	0.076	1.20	8.8	1.0	30.5	0.781	0.47	9.3	12.1	368.1	0.498	0.49	5.4	1.9	99.1	0.858	0.29
	*Lysine*	29.3	2.5	24.1	0.096	1.09	24.1	2.3	27.0	0.186	0.88	5.1	2.1	115.2	0.879	0.19	21.8	12.4	160.4	0.390	0.78	9.7	1.9	54.9	0.858	0.34
	*Methionine*	7.6	0.5	20.0	0.007	2.82	4.9	0.4	23.9	0.027	1.67	1.5	0.7	126.1	0.869	0.46	1.7	1.9	319.1	0.365	0.52	1.3	0.7	147.1	0.858	0.39
	*N,N-Dimethylglycine*	0.6	0.1	37.8	0.096	1.01	0.4	0.1	44.0	0.332	0.67	0.2	0.1	152.0	0.879	0.24	0.3	0.4	386.3	0.579	0.44	0.1	0.0	87.4	0.858	0.15
	*Ornithine*	28.0	2.9	29.5	0.007	2.42	24.5	2.8	32.3	0.012	1.98	12.8	1.7	37.6	0.461	0.91	15.8	8.4	150.9	0.166	1.18	13.7	1.9	40.0	0.692	0.93
	*Phenylalanine*	12.1	0.4	10.1	0.018	2.02	10.3	0.7	20.4	0.031	1.76	6.8	0.4	18.3	0.416	1.06	7.9	3.7	132.7	0.166	1.18	7.8	1.1	38.6	0.174	1.17
	*Proline*	41.5	4.5	30.6	0.142	1.01	33.9	5.8	48.3	0.126	0.82	11.4	3.1	77.5	0.869	0.25	23.0	27.5	337.8	0.390	0.53	13.0	2.9	62.9	0.858	0.29
	*Serine*	36.1	3.5	27.2	0.040	1.54	37.2	3.0	22.6	0.031	1.58	19.5	3.3	47.6	0.593	0.77	32.0	14.2	125.4	0.166	1.27	20.4	2.2	29.9	0.812	0.78
	*Taurine*	13.2	4.6	97.9	0.083	0.94	91.0	5.5	17.1	0.002	6.07	87.4	5.7	18.3	0.004	6.08	80.6	4.5	15.9	0.004	5.14	79.2	4.9	17.5	0.008	6.93
	*Threonine*	24.7	2.7	31.1	0.031	1.02	20.4	2.8	38.5	0.076	0.85	4.1	2.9	201.2	0.928	0.15	13.4	10.2	215.6	0.291	0.51	6.9	2.0	80.6	0.858	0.26
	*Tryptophan*	−0.6	0.3	−164.7	0.672	−0.48	1.4	0.4	87.1	0.086	1.16	0.7	0.4	168.9	0.696	0.74	1.2	0.6	141.9	0.166	1.22	0.8	0.4	166.7	0.812	0.74
	*Tyrosine*	11.6	0.7	17.6	0.040	1.37	9.4	0.9	27.8	0.061	1.10	3.3	0.5	44.6	0.869	0.36	5.2	6.0	327.1	0.390	0.57	4.2	1.0	65.6	0.812	0.44
	*Valine*	51.4	3.7	20.1	0.018	1.64	38.6	3.1	22.3	0.086	1.18	12.8	2.5	55.3	0.869	0.36	16.6	20.4	348.6	0.513	0.46	15.4	3.6	66.0	0.858	0.42
Ketone bodies	*3-Hydroxybutyrate*	16.0	5.0	88.3	0.329	0.36	14.1	5.2	103.1	0.332	0.30	−8.9	8.5	−269.0	0.935	−0.03	−9.0	13.8	−431.7	0.959	−0.05	2.4	4.1	476.1	0.979	0.01
	*Acetoacetate*	2.3	1.8	224.6	0.672	0.24	−28.1	0.7	−7.3	0.027	−1.78	8.2	5.9	−202.2	0.869	−0.22	−8.7	6.7	−217.6	0.700	−0.25	−2.3	0.8	−102.4	0.858	−0.15
	*Acetone*	−13.4	1.4	−29.2	0.115	−1.24	−94.5	5.9	−17.6	0.002	−3.71	−0.2	0.6	−997.6	0.935	−0.03	1.8	3.9	598.3	0.631	0.14	1.3	0.6	126.3	0.858	0.08
Lipid metabolism	*Carnitine*	0.7	1.0	426.3	1.000	0.13	6.2	0.9	41.1	0.031	1.57	−0.3	1.5	−1,298.8	0.999	−0.04	4.7	2.3	136.6	0.166	1.09	0.9	0.6	209.4	0.858	0.19
	*O-Acetylcarnitine*	2.4	0.1	17.4	0.027	1.63	2.0	0.3	34.5	0.086	1.35	0.2	0.5	650.1	0.879	0.17	0.8	0.7	243.8	0.513	0.58	0.2	0.5	573.5	0.979	0.15
Others	*Choline*	1.5	0.2	32.1	0.050	1.20	1.9	0.2	28.9	0.021	1.60	−1.5	2.0	−389.6	0.935	−0.26	0.5	0.9	511.1	0.632	0.38	0.8	0.1	43.2	0.812	0.61
	*Dimethyl sulfone*	1.7	0.3	42.7	0.167	0.87	1.2	0.1	33.2	0.332	0.60	0.0	0.3	3,146.6	0.879	−0.07	0.3	1.0	825.3	0.819	0.14	0.4	0.2	132.6	0.858	0.18
	*Methanol*	−40.2	2.1	−14.8	0.010	−2.06	−1.4	1.1	−220.0	0.751	−0.20	0.1	0.7	3,646.4	1.000	−0.06	−0.1	8.2	−18325.4	0.959	−0.07	0.4	1.4	1,147.7	0.887	−0.08
	*Urea*	1374.6	128.3	26.4	0.007	2.33	1,108.5	115.7	29.5	0.021	1.95	527.3	77.5	41.6	0.461	0.79	424.5	387.6	258.2	0.434	0.62	322.8	84.3	73.9	0.838	0.48
Polyamines and creatine	*Creatine*	7.2	1.3	51.9	0.373	0.43	7.8	1.4	50.2	0.425	0.47	0.4	0.8	551.1	0.928	0.05	4.6	7.9	484.0	0.632	0.28	1.9	0.8	118.8	0.858	0.10
	*Creatinine*	13.8	1.4	27.7	0.115	1.13	10.6	1.0	27.2	0.126	0.83	2.6	0.8	87.5	0.869	0.20	7.0	5.1	206.5	0.365	0.51	2.4	1.4	161.6	0.858	0.18
Pyruvate metabolism	*Lactate*	1285.8	90.8	20.0	0.002	2.91	1129.1	55.3	13.8	0.008	2.60	535.6	76.4	40.3	0.593	1.07	976.5	266.9	77.3	0.087	1.75	623.1	57.5	26.1	0.499	1.25
	*Pyruvate*	−17.4	8.0	−130.9	0.332	−0.82	−8.5	4.3	−143.8	0.549	−0.44	−38.2	5.4	−39.6	0.148	−1.44	66.2	10.0	42.7	0.004	7.60	−4.5	3.4	−210.7	0.935	−0.16
Short-chain fatty acids (SCFAs)	*Acetate*	−1.0	2.5	−718.5	0.815	−0.12	−18.8	2.0	−29.5	0.031	−1.66	−22.5	2.9	−36.6	0.078	−1.85	−13.6	5.3	−110.7	0.343	−1.15	−5.1	1.8	−100.1	0.858	−0.40
	*Butyrate*	1.4	0.2	35.5	0.031	1.85	0.9	0.2	70.0	0.126	1.07	0.4	0.1	97.2	0.781	0.45	−0.5	0.5	−290.0	0.632	−0.45	0.2	0.3	314.2	0.858	0.29
	*Formate*	−123.1	2.0	−4.5	0.002	−20.91	−24.7	0.6	−6.5	0.002	−9.27	−171.4	2.6	−4.3	0.004	−23.60	−1.3	0.7	−144.7	0.506	−0.65	−1.2	0.5	−120.6	0.858	−0.64
Sugars	*Glucose*	−18253.0	338.5	−5.2	0.002	−27.52	377.6	48.4	36.2	0.039	1.22	−200.2	74.0	−104.6	0.869	-0.47	6.7	150.7	6,328.0	0.955	0.04	6.9	69.9	2,858.2	1.000	0.02
	*Mannose*	5.3	1.3	68.5	0.293	0.76	1.4	0.8	154.8	0.878	0.20	−1.7	1.4	−243.3	0.879	−0.26	−1.8	3.4	−547.7	0.768	−0.18	−1.9	0.9	−137.4	0.858	−0.25
	*Myo inositol*	7.2	0.7	26.7	0.003	2.75	7.3	1.0	40.1	0.002	2.97	2.6	1.2	127.3	0.461	0.98	6.7	1.1	48.1	0.010	2.33	1.7	0.5	88.0	0.838	0.62
Tricarboxylic acid (TCA) cycle	*2-Oxoglutarate*	2.4	0.7	80.0	0.096	1.16	1.2	0.7	156.7	0.373	0.52	−0.7	0.7	−260.8	0.869	−0.35	2.6	1.5	164.6	0.191	1.07	0.6	0.3	134.1	0.858	0.24
	*Citrate*	−32701.4	1,031.2	−8.9	0.002	−25.64	−20904.7	566.8	−7.7	0.002	−23.90	−7.7	4.4	−160.0	0.831	−0.26	−14.9	16.8	-320.5	0.365	−0.44	19.5	3.7	53.4	0.812	0.64
	*Succinate*	−2.7	0.5	−50.7	0.065	−1.35	−0.1	0.2	−587.7	0.851	−0.05	0.4	0.3	208.1	1.000	0.12	1.3	1.0	221.8	0.632	0.55	0.8	0.2	87.9	0.858	0.30

a Pairwise comparison between serum (Reference) compared to each plasma type was performed using the Mann-Whitney U test.

b Effect size (δ) between serum and each plasma tube are presented: |δ| < 0.33 corresponds to small, |δ| < 0.474 corresponds to medium, and |δ| > 0.475 corresponds to large effect size in metabolite concentration differences.

## Discussion

Epidemiology has immensely contributed to public health by pinpointing important risk factors (often multifactorial environmental and genetic components) that contribute to disease outcome. Together with metabolomics, epidemiology is now in the position to be able to uncover biological mechanisms to refine the relationship between exposure and disease in humans, which in turn may offer opportunities for intervention. Blood is one of the most banked samples and thus mining and comparing samples between studies requires understanding how metabolite signatures are affected by different matrices and different tube types. Here we investigated differences in the metabolite profile of blood samples collected as serum compared to plasma collected utilizing acid citrate dextrose (ACD), citrate, EDTA, fluoride, and heparin anticoagulants.

Utilizing targeted NMR-based metabolomics analysis, we identified and quantified 52 metabolites, 50 of which were present in all blood samples and were compared. Our results show a high degree of repeatability in terms of sample preparation, data acquisition, and data analysis, showing that the NMR method is precise, and produces highly robust reproducible quantitative data. Historically, serum has been the preferred assay material because it does not require any anticoagulants for its collection. Serum is used to assess clinical chemistry parameters, drug levels, and blood bank procedures, and as such was used as our gold standard. Overall, we found that the analysis of heparin plasma, followed by EDTA, and fluoride plasma had similar metabolic profiles to serum. Heparin, in particular, only differed in 3 of 50 metabolites (arginine, glutamate, and taurine), and had a nearly identical metabolic fingerprint to serum (Figure 1). Previous studies have also found minimal differences between serum and heparin plasma samples (Teahan et al., 2006). EDTA plasma also had a similar metabolic profile to serum. However, EDTA produces strong signals in ^1^H-NMR spectra which could obscure neighboring metabolites, such as choline, dimethylamine, and one signal of citrate (Bernini et al., 2011). Our results agree with previous findings from Barton et al. (2010) who also found EDTA had negligible effects on the overall metabolic fingerprint. Very limited work has been done on fluoride tubes, and we found notably higher levels of pyruvate in serum compared to fluoride plasma. Fluoride tubes are specialized tubes that contain sodium fluoride to inhibit the metabolic processes of glycolysis by erythrocytes. This explains the difference in pyruvate concentration obtained by the analysis of samples from fluoride tubes and serum tubes. Nonetheless, most metabolites in fluoride tubes were very similar to serum tubes including ketone bodies, lipid metabolism metabolites, short-chain fatty acids, tricarboxylic acid cycle intermediates, most amino acids, and sugars.

We found that ACD plasma and citrate plasma were very different from serum, largely due to significant interfering peaks (from citrate and glucose) in the NMR spectra which originate from the anticoagulants themselves. One could exclude glucose and citrate metabolites from analysis to utilize these plasma tube types; however, it comes with the cost of losing the ability to quantify these two important biological compounds. Interestingly, although substantial differences were associated with tube types in the metabolic profiles, clear differences between subjects are preserved, even among ACD and Citrate tubes. For example, in Figure 1, samples from subjects B and E largely cluster together due to high levels of ketone bodies (acetoacetate, acetone, and 3-hydroxybutyrate) regardless of tube type. Indeed, we have successfully utilized ACD plasma samples in a previous study to investigate metabolomic differences among individuals with developmental disabilities in an epidemiological case-control study bridging metabolomics and epidemiology (Orozco et al., 2019). However, we limited our analysis to only include individuals with ACD samples and did not include any serum samples to avoid any confounded results based on measurement errors from the collection tube rather than true biological differences, and we excluded citrate and glucose from our analysis.

An interesting finding in the present study is that most amino acids and their derivatives had higher concentrations in serum compared to all plasma tube types. This finding agrees with previous studies, even across different analytical platforms. For example, Denery et al. (2011) also found higher concentrations of amino acids and their metabolites in serum compared to heparin plasma using liquid chromatography−mass spectrometry (LC-MS). Paglia et al. (2018) found amino acid concentrations were higher in serum compared to EDTA and citrate plasma utilizing LC-MS. Nishiumi and colleagues (Nishiumi et al., 2018) reported higher amino acids and derivatives levels in serum compared to EDTA plasma utilizing LC-MS. Additionally, Yu et al. (2011) also found significantly higher amino acid levels in serum compared to EDTA plasma. One possible explanation for the difference in amino acid concentrations is that the added anticoagulants them-selves are likely to dilute the samples. ACD tubes, for example, contain trisodium citrate (22.0 g/L), citric acid (8.0 g/L), and dextrose (24.5 g/L). Similarly, citrate plasma tubes contain 3.2% buffered sodium citrate solution. Additionally, differences in amino acids could also be due to the coagulation step of serum collection, which is likely to concentrate metabolites in serum in a reduced volume (Paglia et al., 2018). Both of these factors may play a role in the different plasma amino acid levels relative to serum.

Regarding other notable differences in serum compared to plasma, we found that pyruvate, acetate, and formate were significantly lower in serum compared to EDTA plasma. Suarez-Diez et al. (2017) also reported lower concentrations of formate and pyruvate in serum compared to EDTA plasma. Additionally, lactate was notably higher in serum compared to all plasma tubes, though it only reached statistical significance in ACD, Citrate plasma, and Fluoride plasma. Lopez-Bascon et al. (2016) also reported significantly higher lactate in serum compared to EDTA plasma, while Teahan et al. (2006) found that lactate was higher in serum compared to heparin plasma. Lopez-Bascon also found higher concentrations of myo-inositol in serum compared to EDTA plasma. Similarly, we found myo-inositol was higher in serum compared to ACD, citrate plasma, and fluoride plasma.

Overall, there are important aspects that should be considered when designing an experiment where metabolomics analysis might be performed, preparing to bank samples, or using banked samples. We recommend the use of serum for metabolomics studies since anticoagulants that interfere with downstream laboratory applications are avoided, and the impact of these anticoagulants on the concentration of certain metabolites, such as amino acids, can be avoided. In the case that serum is unavailable, we have shown that both heparin plasma and EDTA plasma approximate the concentrations observed in serum closely. Further, we suggest that the mean difference summarized in our study (Tables 1, 2) can be utilized as a correction factor to adjust metabolite concentrations collected in plasma to be similar to concentrations in serum. This could be useful to pool data from biobanked samples across epidemiological studies that were not collected using the same tube. Similarly, corrections could be used for meta-analyses combining results across studies of metabolites collected in different tubes.

Additional considerations in blood tube choice may depend on other intended downstream analyses. Heparin plasma (which inhibits thrombin activity) and EDTA plasma (which binds calcium ions) are both broadly used in clinical and epidemiological research. However, heparin binds to DNA during purification and inhibits Taq polymerase used for polymerase chain reaction (PCR). Although we have shown this is not problematic for NMR-based metabolomics, these samples would not be recommended for DNA work. Likewise, one also needs to consider if the large EDTA peaks in the NMR spectra may interfere with metabolites of interest [such as choline, dimethylamine, and one peak of citrate (Bernini et al., 2011)]. Furthermore, an important distinction also needs to be made between conventional blood collection tubes and those utilizing gel separator tubes for investigators considering metabolomics analysis. Gel separator tubes are used to accelerate the process of serum or plasma separation and theoretically should not change the metabolite composition because of the inertness of gel. Yet, several studies have shown changes in the metabolite fingerprints of samples collected utilizing polymeric gel tubes compared to conventional tubes, particularly for amino acids (Yu et al., 2011; López-Bascón et al., 2016). As such, the use of gel separator tubes is not recommended.

We have chosen to utilize serum as the gold standard in our study by which to compare all other plasma tubes due to the broader applications of serum, the limitations of some anticoagulants in plasma, and because previous studies have found that serum samples had the greatest number of recovered metabolites compared to plasma (Denery et al., 2011; Cruickshank-Quinn et al., 2018; Nishiumi et al., 2018). However, a limitation of serum is that the processing time can be subject-dependent (i.e., clotting time may vary across individuals) (Tuck et al., 2009). Therefore, metabolic processes from biologically active analytes may still be occurring and affect accurate metabolite quantification in serum. Other studies have shown pre-analytical steps, such as freeze-thaw cycles, can negatively affect the metabolome profile (Bernini et al., 2011; Townsend et al., 2016; Cruickshank-Quinn et al., 2018; Nishiumi et al., 2018). A strength in our study is that aliquots of all samples were immediately frozen at -80°C after collection, were under the same storage duration and conditions, and never underwent previous freeze-thaw cycles before NMR-based metabolomics analysis, which could have negatively affected metabolite stability.

## Conclusion

Careful consideration about which blood collection matrix to use in a study is critical to obtain meaningful biological inferences from metabolome data. While serum is considered the gold standard, we have shown that Heparin and EDTA plasma are comparable to serum for NMR-based metabolomics studies. We also found that ACD plasma and citrate plasma were the most different from serum tubes, largely due to significant interfering peaks (from citrate and glucose). Yet, despite the differences in metabolite concentration based on tube type (particularly for ACD and Citrate plasma), clear differences between subjects were preserved regardless of tube types. Our results, and others, show serum samples have higher levels of amino acids and their derivatives compared to plasma. Bridging technological advancements in metabolomics with classical epidemiological approaches can provide new insight into the etiology of diseases.

## Data Availability

The original contributions presented in the study are included in the article/Supplementary Material, further inquiries can be directed to the corresponding author.
