# Supplementary material for: A Comparison of Serum and Plasma Blood Collection Tubes for the Integration of Epidemiological and Metabolomics Data
Source: Front Mol Biosci. 2021 Jul 8;8:682134. doi: 10.3389/fmolb.2021.682134 (PMC8295687; doi:10.3389/fmolb.2021.682134)
Supplement: Supplementary file 1 [file DataSheet1.PDF]

Supplementary Material

1 Supplementary Figures and Tables

1.1 Supplementary Figures

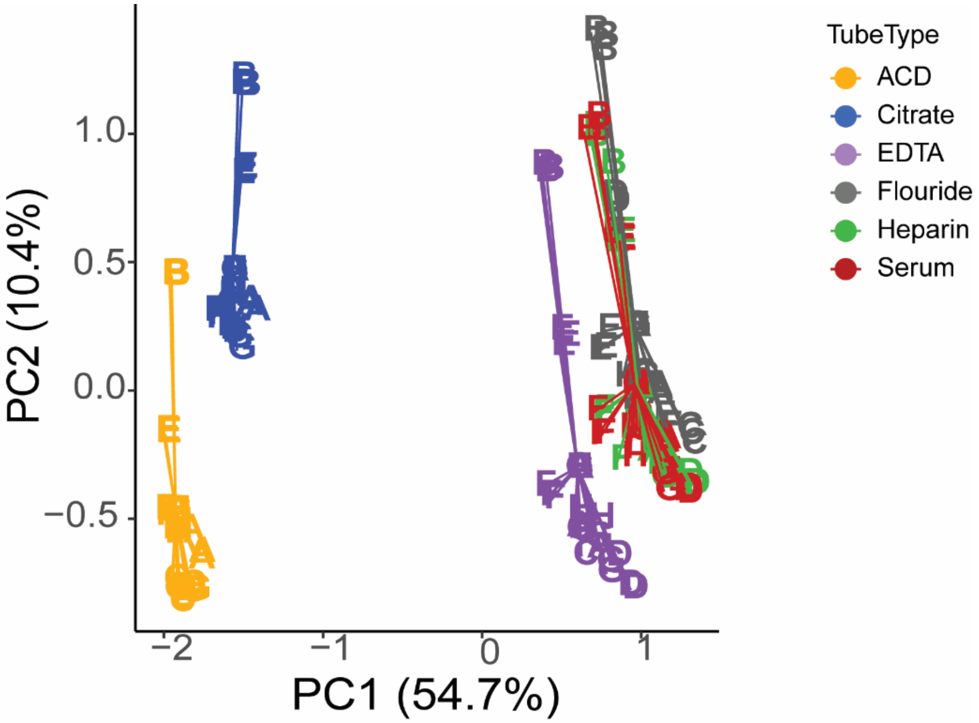

Supplementary Figure 1. NMR experiment run in triplicate. PCA plot showing overlap between each replicate of the NMR experiment. Principal component 1(PC1) accounted for 54.7% of variation and PC2 accounted for 10.4% of the variation. Replicates are denoted by letters of the same color (i.e. tube type) where yellow=ACD; blue=citrate, lavender=EDTA, Fluoride = gray, Heparin=green, and Serum=red. For example, three grey "B"s top right show the three separate ^{1}H NMR runs of Fluoride samples (gray) of Subject B.

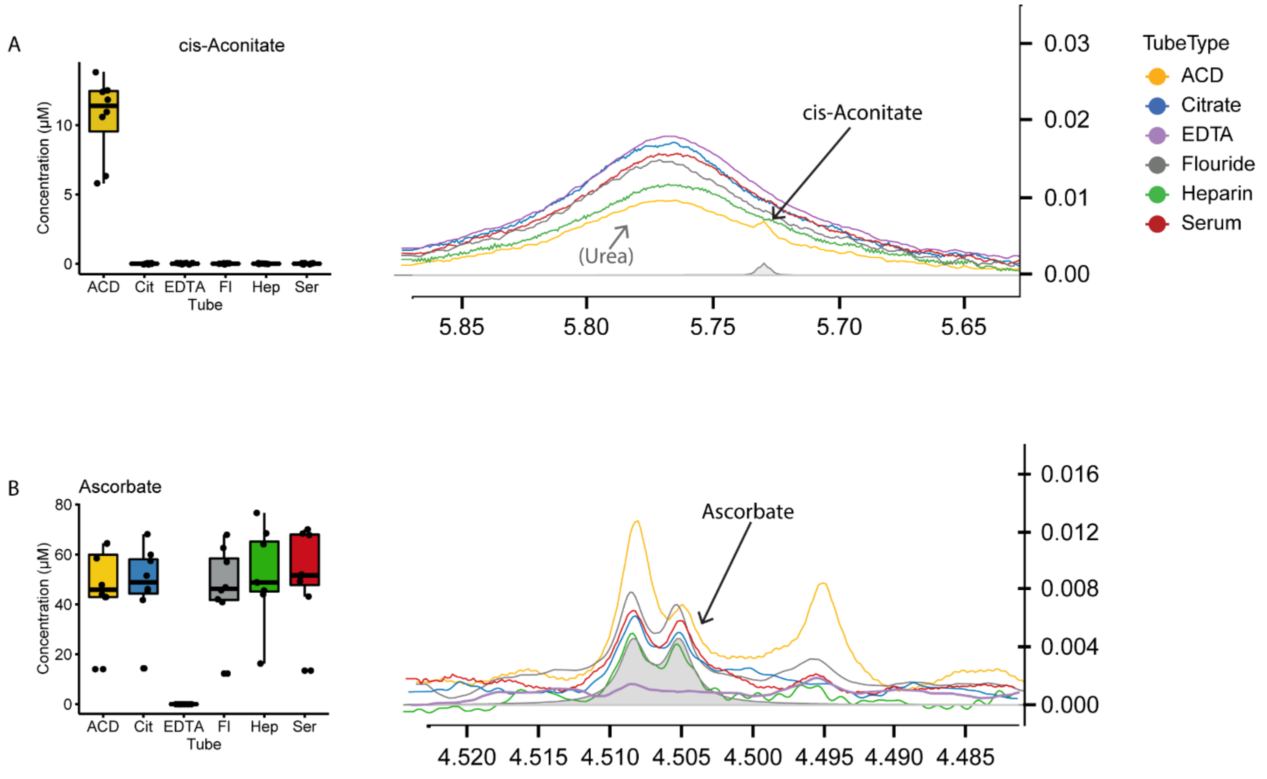

Supplementary Figure 2. Metabolites excluded. Metabolites which were not present in all tube types and excluded from any analysis: A) Cis-Aconitate, B) Ascorbate. Left panel shows box-and-whisker plots, and NMR spectra on right panel for each metabolite.

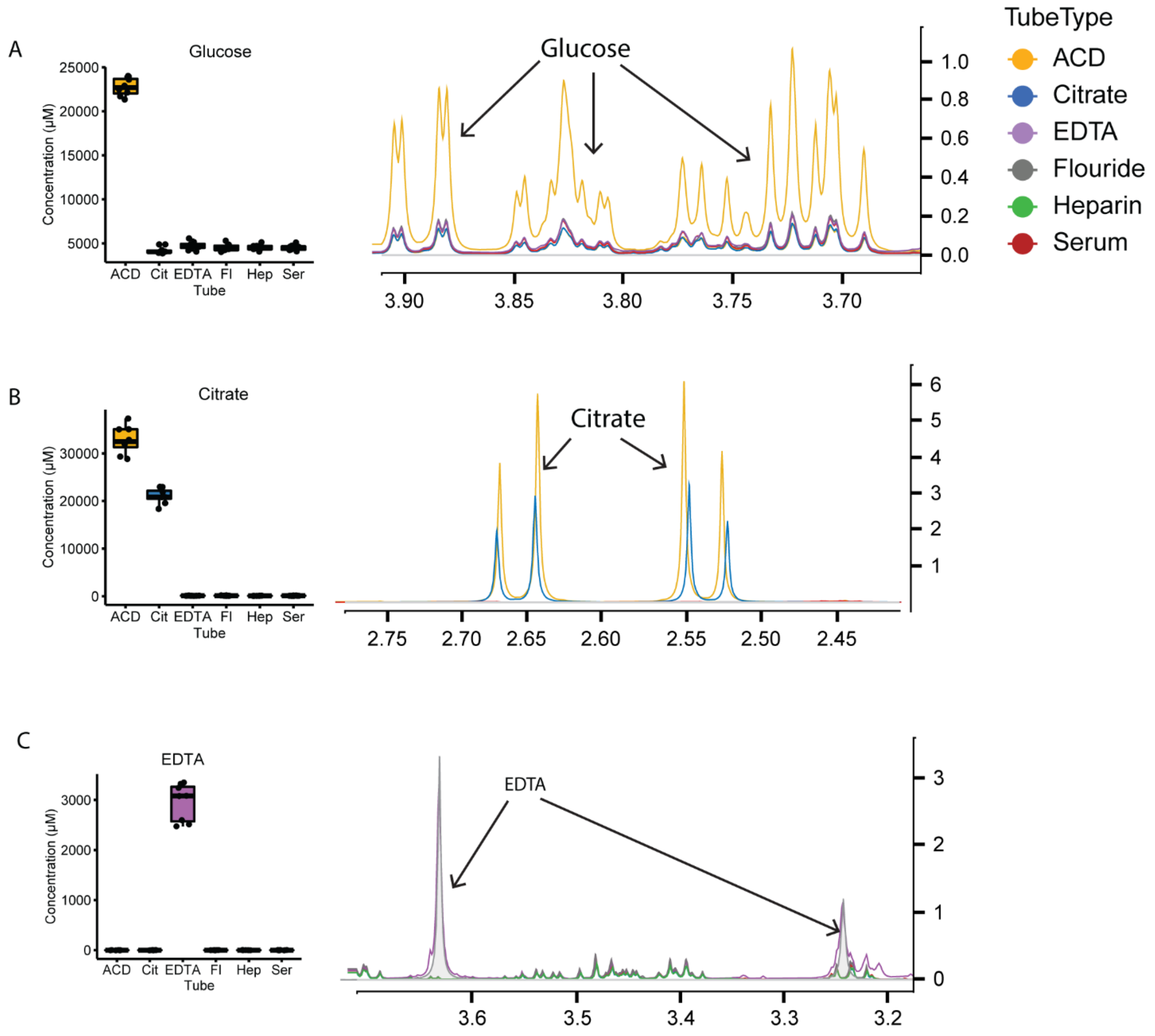

Supplementary Figure 3. Metabolite additives in plasma tube types. A) Glucose in ACD plasma, B) Citrate in ACD and citrate plasma, and C) EDTA in EDTA plasma. Left panel shows box-and-whisker plot, right panel shows NMR spectra for each metabolite.

1.2 Supplementary Tables

Supplementary Table 1. Summary of extant literature. Current findings from metabolomics studies comparing blood collection tube types compared to the present study (highlighted in gray at the top). Other studies are arranged by date/author.

Samples/ Subjects	Collection Tubes	Analytical platform[a]	Key takeaway/Metabolite differences	Reference
8 healthy fasted volunteers	Serum= no additive<br><br>Plasma= ACD, Citrate, EDTA, Fluoride, and Heparin	NMR	• Amino acid concentrations higher in serum compared to all plasma tube types<br>• Serum most like Heparin, and EDTA plasma<br>• Serum very different to ACD and Citrate plasma metabolome	Present Study
4 non-fasted volunteers	Serum= no additive<br><br>Plasma=Heparin	NMR	• Changes in energy metabolites (lactate (increased) and glucose (decreased)) occur after prolonged room temperature exposure for serum<br>• Differences between serum and heparin plasma were minimal<br>• A small increase in triglyceride levels in serum relative to plasma	2006, (Teahan et al., 2006)
36 healthy volunteers	Plasma=EDTA, Heparin	UPLC-MS	• Heparin best anticoagulant for blood collection	(Pereira et al., 2010)
6 healthy adults (3 males, 3 females)	Serum= separator tube (SST)<br><br>Plasma=K_2EDTA	NMR	• Changes in glycolysis metabolites (glucose, lactate, and pyruvate) were affected more in serum tubes. Time changes (0-24 hours) were identified in triglycerides, proline, choline, citrate, and histidine in serum more than plasma<br>• Degradation processes are time-dependent and temperature-dependent both for serum and plasma samples at 4C and 25C<br>• EDTA or citrate plasma recommended for NMR-based metabolomics analysis	(Bernini et al., 2011)
12 healthy volunteers	Serum= no additive<br><br>Plasma= Heparin	LC-MS	• Peptide/peptide fragments were found in higher concentration in serum<br>• Only lysophosphatidylinositol was found to be higher in the plasma<br>• Serum may be more beneficial for small-molecule analysis	(Denery et al., 2011)
29 small-cell lung cancer (SCLC) patients	Serum= no additive<br><br>Plasma= Li-Heparin	GC-TOF-UPLC/MS	• Variation among subjects was found to be similar between serum and plasma for the majority of metabolites<br>• However, tryptophan, galactose, malonic acid, leucine, and serine showed more variation among subjects' serum than in plasma. While α-Hydroxybutyric acid and tyrosine showed more variation among subjects' plasma than in serum.<br>• Glycerophosphocholines, erythritol, creatinine, hexadecanoic acid, and glutamine in plasma, but not in serum, were shown to correlate with life expectancy for SCLC patients, indicating the utility of metabolomics analyses in clinical prognosis	(Wedge et al., 2011)
377 individuals (197 males, 180 females)	Serum= no additive<br><br>Plasma= EDTA gel tubes	AbsoluteIDQ kit 150- FIA-MS	• Metabolite profiles from plasma and serum were distinct with 104 metabolites showing significantly higher concentrations in serum<br>• More unique metabolites were identified in serum than in plasma<br>• Reproducibility is good for both plasma and serum, and better in plasma<br>• Higher metabolite concentrations were found in serum	(Yu et al., 2011)
3 healthy volunteers	Serum= no additive<br><br>Plasma= Li-Heparin, sodium fluoride, sodium citrate, potassium EDTA	LC-MS	• Anticoagulant choice affected the ionization process of LC-MS<br>• Recommend use of EDTA plasma collection tubes	(Yin et al., 2013)

13 healthy volunteers (3 men, 10 women)	Serum= plastic spray-coated silica, plastic spray-coated silica with a polymer gel (serum-gel)<br><br>Plasma= spray-coated silica tubes with heparin, Heparin with polymer gel (plasma-gel)	GC-TOF/MS	• Serum samples had the greatest number of metabolites found<br>• Differences between plasma and serum were found affecting citric acid cycle, metabolism of amino acids, the fructose and mannose metabolism, and that of glycerolipids and pentose/glucuronate interconversion<br>• In general, samples collected in gel-separator tubes reported a lower number of metabolites. Significant changes attributable to the polymeric gel were detected in serum only	(López-Bascón et al., 2016)
377 individuals (197 males, 180 females)	Serum= No additive<br><br>Plasma=EDTA plasma gel tubes	AbsoluteIDQ kit 150-NMR	• 23 out of 29 metabolites had lower levels in plasma compared to serum<br>• only formate, pyruvate, alanine, threonine and oxoglutarate concentrations were significantly higher in plasma than serum<br>• no significant difference was found for 3-hydroxybutyrate<br>• From a network-analysis prospective, plasma and serum metabolite networks possess the same general characteristics, though differences arise as in the case of amino acids, and should be taken into account when analyzing, comparing and interpreting blood metabolite association networks	(Suarez-Diez et al., 2017)
12 week-old male Wistar rats	Serum=gel separator<br><br>Plasma=EDTA-K2, heparin lithium, heparin sodium, sodium citrate, and potassium oxalate	UPLC/MS	• Heparin plasma had the best detection of different functional groups and was therefore recommended as optimal blood specimen for metabolomics analysis, followed by potassium oxalate plasma	(Zhou et al., 2017)
3 healthy volunteers (2 males, 1 female)	Serum= SST<br><br>Plasma=EDTA and P100 plasma	LC-MS	• Over 93% of metabolites identified were consistent across tube types. However, serum samples had a higher number and more unique metabolites compared to plasma samples<br>• Metabolites were more sensitive to tube differences (particularly lipids) compared to processing times	(Cruickshank-Quinn et al., 2018)
10 healthy volunteers (5 males, 5 females)	Serum= No additive<br><br>Plasma=K+EDTA	UPLC/MS	• 216 identified metabolites overall: 99 were significantly different between plasma and serum (including acylcarnitines, free fatty acids, lysophospholipids, lysophosphatidylethanolamines, phosphatidylcholines, phosphatidylethanolamines, ether phospholipids, and sphingomyelins)<br>• Only 3 metabolites (carnitine C2:0, carnitine C3:0, and methionine) were significantly lower in serum vs plasma<br>• No significant differences were observed for most amino acids<br>• Recommend use of plasma	(Liu et al., 2018)
3 healthy volunteers	Serum= clot activating and separating gel<br><br>Plasma-EDTA-2Na	LC-MS, GC-MS	• 76 metabolites concentrations significantly different between plasma and serum<br>• Most carbohydrates, amino acids, and their derivatives exhibited higher levels in serum than in plasma<br>• Pre-centrifugation storage time (0 vs 30 min) significantly affected the plasma level of 45 metabolites	(Nishiumi et al., 2018)
80 participants (40 healthy young <35yrs, 40 healthy elderly >60)	Serum= No additive<br><br>Plasma=EDTA, Citrate	LC-MS/ MS AbsoluteIDQ p180 Kit Biocrates	• Serum concentration levels were higher compared to citrate and EDTA, in particular for amino acids and biogenic amines<br>• Blank EDTA tubes contain a significant amount of sarcosine<br>• The highest number of metabolites significantly changing with ages was detected in serum<br>• Recommend serum samples for biomarker discovery studies	(Paglia et al., 2018)

[a]Abbreviations: **GC-TOF-UPLC/MS** : gas chromatography/time-of-flight mass spectrometry and ultrahigh-performance liquid chromatography mass spectrometry; **UHPLC-MS/MS**: ultrahigh-performance liquid chromatography mass spectrometry; **NMR**-nuclear magnetic resonance; **LC-MS**: liquid chromatography−mass spectrometry.

References for Supplementary Table 1.

Bernini, P., Bertini, I., Luchinat, C., Nincheri, P., Staderini, S., and Turano, P. (2011). Standard operating procedures for pre-analytical handling of blood and urine for metabolomic studies and biobanks. *Journal of Biomolecular NMR* 49(3)**,** 231-243. doi: 10.1007/s10858-011-9489-1.

Cruickshank-Quinn, C., Zheng, L.K., Quinn, K., Bowler, R., Reisdorph, R., and Reisdorph, N. (2018). Impact of Blood Collection Tubes and Sample Handling Time on Serum and Plasma Metabolome and Lipidome. *Metabolites* 8(4). doi: 10.3390/metabo8040088.

Denery, J.R., Nunes, A.A.K., and Dickerson, T.J. (2011). Characterization of Differences between Blood Sample Matrices in Untargeted Metabolomics. *Analytical Chemistry* 83(3)**,** 1040-1047. doi: 10.1021/ac102806p.

Liu, X., Hoene, M., Wang, X., Yin, P., Haring, H.U., Xu, G., et al. (2018). Serum or plasma, what is the difference? Investigations to facilitate the sample material selection decision making process for metabolomics studies and beyond. *Analytica Chimica Acta* 1037**,** 293-300. doi: 10.1016/j.aca.2018.03.009.

López-Bascón, M.A., Priego-Capote, F., Peralbo-Molina, A., Calderón-Santiago, M., Luque De Castro, M.D., Lopez-Bascon, M.A., et al. (2016). Influence of the collection tube on metabolomic changes in serum and plasma. *Talanta* 150**,** 681-689. doi: 10.1016/j.talanta.2015.12.079.

Nishiumi, S., Suzuki, M., Kobayashi, T., and Yoshida, M. (2018). Differences in metabolite profiles caused by pre-analytical blood processing procedures. *Journal of Bioscience and Bioengineering* 125(5)**,** 613-618. doi: https://doi.org/10.1016/j.jbiosc.2017.11.011.

Paglia, G., Del Greco, F.M., Sigurdsson, B.B., Rainer, J., Volani, C., Hicks, A.A., et al. (2018). Influence of collection tubes during quantitative targeted metabolomics studies in human blood samples. *Clinica Chimica Acta* 486**,** 320-328. doi: https://doi.org/10.1016/j.cca.2018.08.014.

Pereira, H., Martin, J.-F., Joly, C., Sébédio, J.-L., and Pujos-Guillot, E. (2010). Development and validation of a UPLC/MS method for a nutritional metabolomic study of human plasma. *Metabolomics* 6(2)**,** 207-218. doi: 10.1007/s11306-009-0188-9.

Suarez-Diez, M., Adam, J., Adamski, J., Chasapi, S.A., Luchinat, C., Peters, A., et al. (2017). Plasma and Serum Metabolite Association Networks: Comparability within and between Studies Using NMR and MS Profiling. *Journal of proteome research* 16(7)**,** 2547-2559. doi: 10.1021/acs.jproteome.7b00106.

Teahan, O., Gamble, S., Holmes, E., Waxman, J., Nicholson, J.K., Bevan, C., et al. (2006). Impact of analytical bias in metabonomic studies of human blood serum and plasma. *Analytical chemistry* 78(13)**,** 4307-4318.

Wedge, D.C., Allwood, J.W., Dunn, W., Vaughan, A.A., Simpson, K., Brown, M., et al. (2011). Is serum or plasma more appropriate for intersubject comparisons in metabolomic studies? An assessment in patients with small-cell lung cancer. *Analytical Chemistry* 83(17)**,** 6689-6697. doi: 10.1021/ac2012224.

Yin, P., Peter, A., Franken, H., Zhao, X., Neukamm, S.S., Rosenbaum, L., et al. (2013). Preanalytical aspects and sample quality assessment in metabolomics studies of human blood. *Clinical Chemistry* 59(5)**,** 833-845.

Yu, Z., Kastenmüller, G., He, Y., Belcredi, P., Möller, G., Prehn, C., et al. (2011). Differences between Human Plasma and Serum Metabolite Profiles. *PloS One* 6(7)**,** e21230-e21230. doi: 10.1371/journal.pone.0021230.

Zhou, Z., Chen, Y., He, J., Xu, J., Zhang, R., Mao, Y., et al. (2017). Systematic evaluation of serum and plasma collection on the endogenous metabolome. *Bioanalysis* 9(3)**,** 239-250. doi: 10.4155/bio-2016-0078.
